# A Dedicated Stitch to Allow Early Safe Mobilization Avoiding Drain-Induced Heart Injury

**DOI:** 10.21470/1678-9741-2019-0289

**Published:** 2019

**Authors:** Walter J. Gomes, Isadora S. Rocco, Caroline Bublitz, Isis Begot, Marcela Viceconte, Walace de Souza Pimentel, Nelson Hossne Jr., Alexandre R. Carvalho, Eduardo Gregório Chamlian, Rita Simone L. Moreira, Ross Arena, Solange Guizilini

**Affiliations:** 1 Discipline of Cardiovascular Surgery and Cardiology, Escola Paulista de Medicina, Hospital São Paulo, Universidade Federal de São Paulo - UNIFESP, São Paulo, SP, Brazil.; 2 Hospital Geral de Pirajussara/Associação Paulista para o Desenvolvimento da Medicina - SPDM, Taboão da Serra, SP, Brazil; 3 Hospital de Clínicas Luzia de Pinho Melo/SPDM, Mogi das Cruzes, SP, Brazil.; 4 Department of Physical Therapy, College of Applied Health Sciences, University of Illinois at Chicago, Chicago, IL, USA.; 5 Department of the Human Movement Sciences, Universidade Federal de São Paulo - UNIFESP, Santos, SP, Brazil.

**Keywords:** Coronary Artery Bypass Grafts, Arterial Grafts, Off-Pump Surgery, Drainage, Heart Injury

## Abstract

Placement of a mediastinal drain is a routine procedure following heart surgery. Postoperative bed rest is often imposed due to the fear of potential risk of drain displacement and cardiac injury. We developed an encapsulating stitch as a feasible, effective and low-cost technique, which does not require advanced surgical skills for placement. This simple, novel approach compartmentalizes the drain allowing for safe early mobilization following cardiac surgery.

**Table:** 

Abbreviations, acronyms & symbols	
CABG	= Coronary artery bypass surgery
CR	= Cardiac rehabilitation
ICU	= Intensive care unit

## INTRODUCTION

Mediastinal drain insertion is a routine procedure following cardiac surgery. Although rarely reported, catastrophic complications provoked by mediastinal tubes may occur, such as perforation of cardiac chambers, injuries to the great vessels, damage to coronary grafts, and myocardial ischemia^[1,2]^. Recently, this issue has drawn renewed attention due to the importance of early postoperative mobilization when the mediastinal tube is still in place.

Early mobilization is an important part of the inpatient cardiac rehabilitation (CR) following surgical heart procedures to improve postoperative recovery (*e.g.*, improves cognitive status and functional capacity). Exercise-based, inpatient CR contributes to a reduced incidence of perioperative complications, decreasing intensive care unit (ICU) and postoperative stay as well as reducing future morbidity and mortality in a cost-effective manner^[3]^.

To ensure that the benefits of early mobilization are achieved while minimizing risk to the patient, we developed a novel operative maneuver; a dedicated stitch aimed to compartmentalize and keep the mediastinal drain insulated from the heart chambers.

## TECHNIQUE

This stitch technique was routinely performed on 543 consecutive patients undergoing coronary artery bypass grafting (CABG). In our practice, off-pump CABG comprises >70% of the surgical revascularization procedures. The heart operation is conducted in a usual manner, the pericardium is opened using reversed T incision, alongside the diaphragm. At the completion of the operation, one mediastinal chest tube is inserted behind the sternum through a stab incision in the subxiphoid area. Additionally, pleural drains are placed as needed, coming out through separate incisions in the subxiphoid region. The drains are connected to a vacuum suction system at 15 cmH_2_O negative pressure.

**Fig. 1:**
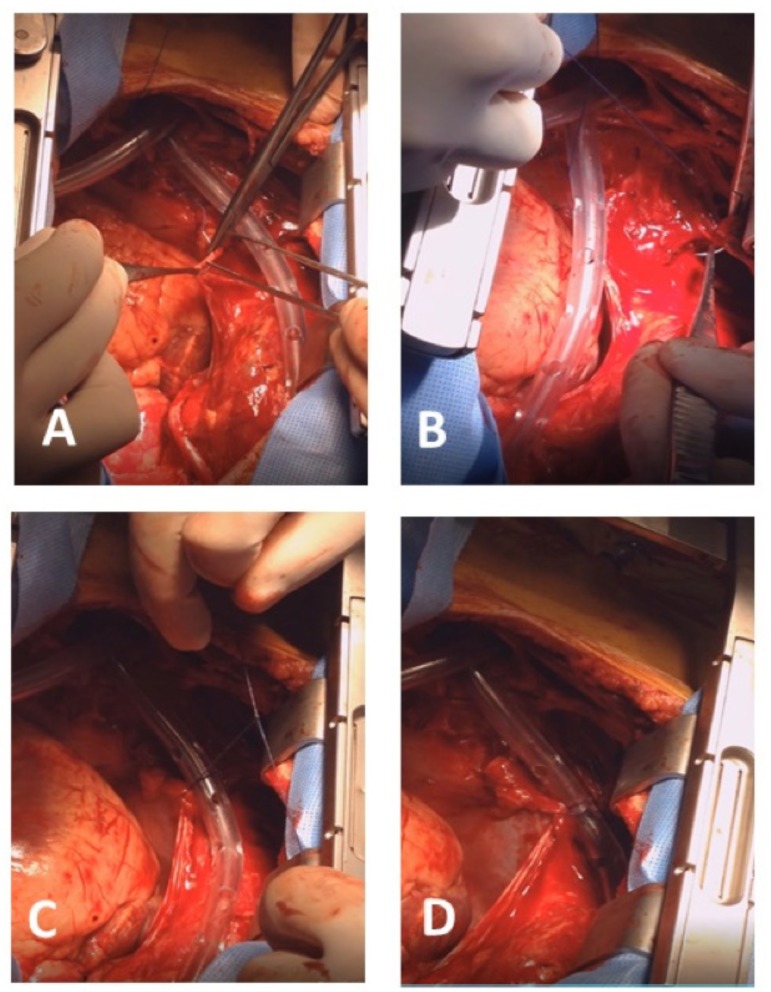
A - A 4-0 polypropylene stitch is applied to the lower medial edge of the right pericardium; B - Sutured to the tissue immediately below the sternum; C - Suture being loosely tied down; D - Drain encapsulated and separated from contact with the heart chambers.

To avoid the mediastinal drain lying directly over the heart, the right aspect of the pericardium is folded around the tube, providing insulation from the heart. To accomplish this, a 4-0 polypropylene stitch is applied to the lower medial edge of the right pericardium (Figure 1A) and sutured to the tissue immediately below the sternum (Figures 1B and 1C), encapsulating the drain and separating it from contact with the heart chambers (Figure 1D); we have named this technique “*the safe stitch*” (Video 1).

**Video 1:**
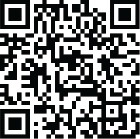
The technique for the "safe stich" placement.

The sternum is closed in a usual manner following drain positioning and confirming stability. All patients were placed in upright position during the first postoperative day before drain removal to evacuate any residual fluid in the pericardial or pleural cavity. The mediastinal tube was removed in a usual manner when the drain flow rate is reduced to approximately zero until the second postoperative day.

## DISCUSSION

Contact between the mediastinal drain and heart chambers is a common concern in surgical practice. Beyond the risk of drain displacement and cardiac injury, the vacuum-suction system may stick the drain onto the anterior wall of the right ventricle or to bypass grafts, causing tissue lesions. Although uncommonly described, structural damages could be provoked by drain material in proximity to heart tissue. Complications, such as perforation of the right heart chamber, injuries to coronary grafts or to the great vessels, and myocardial ischemia, have been reported^[1,2]^. Softer and more flexible drains could help to avoid those lesions. However, it does not solve the issue of contact between drain and heart chambers^[4]^.

Traditionally, in order to reduce the risk of drain displacement, the surgeon and ICU staff are reluctant to place a patient in the upright sitting position for the first hours following surgery. Conversely, early mobilization, a primary component of inpatient CR, has been shown to be effective in reducing postoperative pulmonary complications, reestablishing functional capacity, and decreasing length of ICU stay and postoperative hospitalization days. Studies have shown that early initiation of inpatient CR, including postural changes, during the first postoperative days, is crucial to enhance recovery following cardiac surgery^[3,5]^.

Early removal of mediastinal and pleural drains could be an alternative to reduce one of the hindering factors to early initiation of inpatient CR. However, Andreasen et al.^[6]^ demonstrated that early removal of drains potentially increases the incidence of effusions evolving and the subsequent necessity of further corrective invasive procedures. Therefore, to avoid such postoperative complications, drain removal should respect the proper removal of residual fluid in the pleural or pericardial cavity.

To meet the unique challenges of the cardiac surgery’s postoperative period, allowing for simultaneous preservation of drain placement and early mobilization, we developed *the safe stitch* to keep the tubular drain away from the heart chambers and the right coronary graft, which is usually close to the mediastinal drain. The drain removal maneuver is harmless and performed as usual. The additional leverage of *the safe stitch* technique allows for early mobilization strategies with postural changes. Moreover, *the safe stitch* technique is simple, quickly accomplished, and does not require sophisticated and expensive materials.

*The safe stitch* technique initiated at our institution has allowed for early mobilization; all patients are placed in an orthostatic sitting position early in the first postoperative day. Later, still in the first postoperative day, an exercise-based CR protocol is initiated, composed of walking and active limb movement.

There has been no incidence of arrhythmias during or immediately after the early initiation of an inpatient CR program in these patients. Bleeding was continuously recorded from the time of sternal closure in the operating room until the drains were removed, usually in the second postoperative day. There was no need for surgical revision due to drain-induced bleeding. No other complications, such as pneumothorax or mediastinal drain displacement, have occurred.

In conclusion, *the safe stich* technique for drain compartmentalization has allowed for early safe mobilization, initiated early in the first postoperative day, without the occurrence of adverse event related to the mediastinal drain. As such, we recommend that *the safe stitch* technique should become a routine procedure following open heart surgery.

**Table:** 

Author's roles & responsibilities			
WJG	Substantial contributions to the conception or design of the work; or the acquisition, analysis, or interpretation of data for the work; drafting the work or revising it critically for important intellectual content; agreement to be accountable for all aspects of the work in ensuring that questions related to the accuracy or integrity of any part of the work are appropriately investigated and resolved; final approval of the version to be published.	ARC	Substantial contributions to the acquisition of data for the work; final approval of the version to be published.
ISR	Drafting the work or revising it critically for important intellectual content; final approval of the version to be published.	EGC	Substantial contributions to the acquisition of data for the work; final approval of the version to be published.
CB	Drafting the work or revising it critically for important intellectual content; final approval of the version to be published.	RSLM	Final approval of the version to be published
IB	Substantial contributions to the acquisition of data for the work; final approval of the version to be published.	RA	Substantial contributions to the revising it critically for important intellectual content; agreement to be accountable for all aspects of the work in ensuring that questions related to the accuracy or integrity of any part of the work are appropriately investigated and resolved; final approval of the version to be published
MV	Substantial contributions to the acquisition of data for the work; final approval of the version to be published.	SG	Substantial contributions to the conception or design of the work; or the acquisition, analysis, or interpretation of data for the work; drafting the work or revising it critically for important intellectual content; agreement to be accountable for all aspects of the work in ensuring that questions related to the accuracy or integrity of any part of the work are appropriately investigated and resolved; final approval of the version to be published
WSP	Substantial contributions to the acquisition of data for the work; final approval of the version to be published.		
NH	Substantial contributions to the acquisition of data for the work; final approval of the version to be published.		
